# Appetite and Subsequent Food Intake Were Unaffected by the Amount of Sourdough and Rye in Soft Bread—A Randomized Cross-Over Breakfast Study

**DOI:** 10.3390/nu10111594

**Published:** 2018-10-30

**Authors:** Kia Nøhr Iversen, Daniel Johansson, Carl Brunius, Thomas Andlid, Roger Andersson, Maud Langton, Rikard Landberg

**Affiliations:** 1 Department of Biology and Biological Engineering, Food and Nutrition Science, Chalmers University of Technology, 412 96 Gothenburg, Sweden; carl.brunius@chalmers.se (C.B.); thomas.andlid@chalmers.se (T.A.); rikard.landberg@chalmers.se (R.L.); 2 Department of Molecular Sciences, Swedish University of Agricultural Sciences, SE-750 07 Uppsala, Sweden; daniel.p.johansson@slu.se (D.J.); Roger.Andersson@slu.se (R.A.); maud.langton@slu.se (M.L.)

**Keywords:** sourdough, rye, appetite, satiety

## Abstract

Sourdough fermented bread has been suggested to have beneficial health effects, in part mediated by increased satiety in the postprandial phase, but only limited research has been conducted to verify this. The current study aimed to investigate the effect of the amounts of sourdough and rye in soft bread on postprandial appetite. On 6 occasions, 23 healthy volunteers consumed 5 different test breads, with varying amount of rye and sourdough, and a yeast-fermented refined wheat control bread as part of a breakfast meal. The sourdough ranged between 9–51% of dough weight and rye content between 35–48% of flour weight. Appetite was recorded using visual analogue scales from immediately before breakfast and every 30 min the following 4 h. An ad libitum lunch was served 4 h after the breakfast meal, from which voluntary energy intake was measured. While some of the test breads resulted in lower hunger ratings and increased sense of fullness compared to the refined wheat bread, there were no differences between the test breads. The content of rye in the test breads differed within a narrow range, which might explain the lack of a consistent effect of rye on appetite. Microstructural examination of the test breads showed an increased aggregation of proteins in the breads with high content of sourdough, indicating additional changes to the breads, beyond change in pH, which may counteract the potential effect of decreased pH in the bread on appetite. In conclusion, our study does not support an effect of sourdough on appetite and ad libitum food intake.

## 1. Introduction

Overconsumption of foods and beverages and subsequent excessive energy intake lead to overweight and obesity, which are important components of many lifestyle-related and metabolic diseases [1,2]. One approach to promote lower individual energy intake could be the development of new foods, which increase satiation and extend feelings of satiety [3]. The regulation of food intake is multifaceted depending on both physiological and cognitive responses as well as environmental cues [4] and even if a meal increases satiety from a physiological perspective, this can be overruled by hedonic factors, such as liking and wanting, as well as environmental triggers such as habitual meal frequency [4,5].

Rye breads have repeatedly been demonstrated to induce satiety and reduce hunger compared to wheat breads [6,7]. This has partially been attributed to the specific content and composition of dietary fiber in rye [8]. Specific characteristics of the food matrix can affect its disintegration during digestion which may have implications for gastric emptying, kinetics of nutrient release and absorption, and in the end, metabolic responses [9]. The dense structure of rye breads, having a food matrix with a continuous starch phase compared with a continuous protein network in wheat breads, has been suggested as an important factor for postprandial insulin responses that could also affect appetite [10,11]. It is therefore important to explore the impact of different ingredients and processing on food structure.

Sourdough fermentation has been hypothesized as a way of producing a healthier bread, potentially through improvement in appetite sensations [12]. The production of organic acids during sourdough fermentation and the subsequent pH lowering in the final product have been hypothesized to reduce gastric emptying rate [13,14]. The effect of acid content in bread have been studied by adding varying amount of acetic acid to bread after baking and increased sensation of satiety as well as reduction in glycemic response after addition of acetic acid to white wheat bread has been shown [15]. However, it is uncertain how acid produced in the bread during sourdough formation affects such responses, as the acid produced during sourdough fermentation, in addition to reducing the pH, might affect the final product in other ways, such as altering the structure. Addition of sourdough to bread was furthermore shown to affect the solubility of fiber and the digestibility of starch, which potentially can affect the satiety response following consumption [16].

In contrast, there are also studies which did not support this potential effect of sourdough in reducing appetite response. A study comparing a yeast-fermented bread to a bread made with a propionate rich sourdough found no effect on appetite ratings or subsequent food intake [17]. Similarly, a study comparing the gastric emptying rate after consumption of a sourdough fermented endosperm rye bread and a wheat bread produced with yeast found no difference in gastric emptying rate between the test breads [18]. Gastric emptying has been proposed as a potential mediator of improved satiety upon consumption of sourdough fermented bread.

The current study was conducted to investigate the effect of breads with different contents of rye and sourdough on subjective appetite ratings as a primary outcome, and subsequent ad libitum food intake as a secondary outcome. Breads with different content of sourdough and proportion of whole grain rye were compared against each other and with a refined yeast-fermented wheat bread (control). No studies have yet compared breads with different amounts of sourdough and rye. Such data is crucial to improve our understanding of the role of sourdough in appetite regulation and can in a longer-term help identify future directions for the development of healthy rye breads.

## 2. Materials and Methods

### 2.1. Study Design

The study was designed as a six-way, randomized, cross-over meal study. In total, 23 volunteers consumed 5 test breads and a refined wheat control bread in randomized order, following a Latin square design, as part of a breakfast meal on 6 separate occasions, at least one week apart. On the day before each test, participants were instructed to avoid vigorous physical activity, alcohol, and high-fiber foods. Participants arrived fasted (≥12 h) at the Department of Molecular Sciences at Uppsala BioCentrum, Ultuna, in the morning between 7:30–8:00. Upon arrival they were served breakfast, as well as an ad libitum lunch meal 4 h later. Participants answered questions about their appetite every 30 min, starting right before consumption of breakfast, until right before consumption of the lunch. Additionally, participants filled in a palatability questionnaire after consuming the breakfast.

Participants were allowed to return to their everyday work/activities during the time in between breakfast and lunch, but were instructed to refrain from any vigorous physical activity during that time and to refrain from consumption of any food or beverages.

The study was conducted under the guidelines laid down in the Declaration of Helsinki, all participants gave informed consent in writing. Since none-invasive methods were used and because no subject data could be traced back to a specific individual, the study did not need any approval from the local ethics review board. All meal tests were conducted in May–June 2016.

### 2.2. Subjects

Males and females between the ages of 20–70 years, with a body mass index (BMI) of 18–25 kg/m^2^ were invited to participate in the study. Participants were recruited through emails sent to employees and students at the Swedish University of Agricultural Sciences, Uppsala. Exclusion criteria were regular use of tobacco, performance of sports on an elite level, weight change of more than 10% of current bodyweight within the past 6 months, as wells as pregnancy, planning of pregnancy within the duration of the study, and lactation. Participants were only included if they were used to eating gluten, dairy, and foods of animal origin. Furthermore, participants were required to have a regular meal pattern, with regular consumption of breakfast, lunch and dinner. Before inclusion, participants completed a three factor eating questionnaire (TFEQ-R21) [19] to ensure they were not prone to exhibit extreme dietary restraints or abnormal eating patterns. For practical reasons and due to time restraints, it was not possible to consider the menstrual cycle of females participating in the study.

Due to the appearance of the breads and the nature of the questions, it was not possible to blind the participants to the difference between rye breads and the wheat control bread. However, the participants were not aware that the amount of rye and sourdough differed between the rye breads.

### 2.3. Breakfast Meals

The test breads and refined wheat control bread were served as part of a breakfast meal consisting of 100 g bread with 15 g margarine and 20 g cheese, 100 g orange juice, and 150 g coffee or tea. On the first occasion, participants were given the choice of coffee or tea, which was kept the same for the remaining occasions. On request, 10 g of milk (1.5% fat) could be added to the coffee/tea on all occasions. The margarine, cheese, and orange juice amounted to 695 kJ, 10 g carbohydrate, 6 g protein, and 12 g fat.

The five test breads were produced in a factorial design with 2 different levels of sourdough (high or low; 51% vs. 30% of dough weight) and 2 different levels of rye (high or low; 48% vs. 35% of flour weight), as well as a center point with medium content of both sourdough (30% of dough weight) and rye (42% of flour weight) (Table 1). The amount of rye was calculated as the sum of rye added as flour and rye added with the sourdough. All 5 recipes contained 500 g whole grain wheat flour, 50 g baker’s yeast, 40 g malted flour, 40 g honey, 40 g oil, and 40 g salt, in addition to varying amounts of sourdough, rye flour and refined wheat flour as described in Table 1.

Sourdough included in all recipes was made from 52 kg whole grain rye flour mixed with 78 kg water at a temperature of 35 °C. In total, 28 g sourdough culture was added and the mix was incubated at 30 °C for 24 h before use in the baking. The sourdough cultures used consisted of 18 g *Lactobacillus brevis* A6 and 10 g yeast *Saccharomyces cerevisiae* TY08M, which is a natural mutant with increased freeze tolerance, created by classical ultraviolet mutagenesis and selection. The parental wild-type strain *S. cerevisiae* TY08, (GenBank Accession number FJ972212 CBS 11440), used for mutagenesis was originally isolated from the Tanzanian fermented food togwa [20]. TY08 was chosen for a natural high freeze tolerance and for being evolutionary/physiologically different from common Baker’s yeast strains.

Dough was prepared by a professional baker, divided into 90 g pieces that were frozen before fermentation and stored in a −21 °C freezer for four days. The doughs were then thawed at room temperature for 80 min, fermented in a proofing chamber (38 °C and 75% RH) for 60 min and baked in a rotational oven. Baking (15 min) was initiated at 230 °C with 8 s of steam, and then immediately lowered to 180 °C. Breads were then frozen again and stored at −21 °C until the test days. A commercially available refined wheat bread (Pågen AB, Malmö, Sweden) was used as a control. Although this is a yeast fermented bread, it did contain a small amount of sourdough (3%) which was likely added as flavoring agent.

### 2.4. Chemical Analysis

Bread samples were freeze dried and then milled with a cyclone sample mill (Retsch, Haan, Germany). Dietary fiber content was analyzed according to the Uppsala method [21]. Fructan, and starch content were analyzed using a K-FRUC kit [22], and a K-TSTA kit [23], respectively (Megazyme, Bray, Ireland). Crude fat was determined according to the method described in the Official Journal of the European Communities (1984) and protein according to the Kjeldahl method (N × 6.25). Dry matter was determined by drying the samples at 105 °C for 16 h according to the AACC International Approved Methods method 44–15A.

Lactic acid and acetic acid were determined enzymatically (Roche, R-Biopharm, Darmstadt, Germany). Briefly, 10 g of sample was mixed with 100 mL distilled water and homogenized. The mixture was heated to 60 °C and kept there for 5 min and then cooled to room temperature in an ice bath. pH was adjusted to 7 with sodium hydroxide (NaOH) and the mixture put in an ultrasonic bath for 5 min. The samples were then centrifuged and filtered through a 0.2 µ membrane filter and stored at −20 °C until analysis. All samples were analyzed in duplicate (Table 2).

### 2.5. Microscopy

The breads were examined microscopically in order to examine potential structural differences between the breads. Bread samples were embedded as described by Johansson et al. [24] and the embedded samples were cut into 2 µm thick sections with an ultra-microtome (Leica EM UC6, Leica, Vienna, Austria). Sections were stained with Fast Green/Lugol’s solution or Calcofluor White. Fast Green colored proteins green while Lugol’s solution colored starch purple/violet making it possible to distinguish between amylopectin rich areas (beige/brown), and amylose (blue). When stained by calcofluor, cell walls rich in β-glucan appeared blue when examined in exciting light (excitation, 400–410 nm; emission, 455 nm). The stained sections were examined using a Nikon Eclipse Ni-U microscope (Nikon Instruments Inc., New York, NY, USA) and the images were captured with a Nikon Digital Sight DS-Fi2 camera (Nikon Instruments Inc., New York, NY, USA) and processed with the software NIS-Elements BR (Nikon Instruments Inc., New York, NY, USA).

### 2.6. Appetite Assessment

Appetite was measured on a handheld computer (Palm z22; Palm Inc., Sunnyvale, CA, USA), with a program specially designed for appetite assessment that has been validated against traditional pen and paper method [25]. At each time point the questions “How hungry do you feel?”, “How full do you feel?”, and “How strong is your desire to eat?” were presented on a visual analogue scale (VAS) and participants marked their answer on the scale. The scales were anchored with the words “Not at all hungry”/“Extremely hungry”, “Not at all full”/“Extremely full”, and “Not at all strong”/“Extremely strong”. The participants answers were recorded as values between 0 and 100, measured from the left.

The questions were presented one at a time on the screen, and participants did not have access to see previously answered questions, preventing participants from referring to previous questions when answering. Participants were provided with questions on paper and instructed to answer the questions on paper if problems occurred with the computers (8.8% of data points were collected on paper).

### 2.7. Ad Libitum Lunch Meal

The lunch was an ad libitum meal of Swedish hash, which is a mix of potatoes, pork and onions chopped in small pieces and fried, served with pickled beetroots and a glass of water. Each participant was served 600 g Swedish hash fried in 6 g rapeseed oil (per 100g: 863 kJ, 10.4 g fat, 21.6 g carbohydrate, 4.3 g protein), 90 g pickled beet roots and 200 mL water. Participants were instructed to eat until they felt comfortably full and instructed to ask for more food if needed. After the participants had left, leftover food was weighed and recorded.

### 2.8. Palatability

Participants were asked to rate 5 aspects of the breakfast meals on a VAS with words anchored at the end and answers were recorded as a value between 0 and 100 measured from the left. The questions (and anchored words) were; “Taste (bad/good)”, “Smell (not appetizing/appetizing)”, Appearance (not appetizing/appetizing)”, “Off taste (none/much)”, and “How appetizing is it (general impression)? (Not appetizing/appetizing)”.

### 2.9. Statistical Analysis

In SAS software, Proc Mixed procedure ANCOVA models were used to evaluate the difference between bread types, here referred to as diet, on fullness, hunger and desire to eat. A repeated statement was included in all models to consider the cross-over design of the study.

First, a within subject repeated measures ANCOVA (referred to as model A) including subject as random factor and diet, time and occasion as fixed factors and baseline (time 0) as a covariate as well as diet x time and diet x occasion interactions, was applied. Interaction terms and occasion were removed if they were not significant (*p* ≥ 0.05).

Secondly, area under the curve (AUC) of appetite assessment (fullness, hunger and desire to eat) was calculated using the trapezoid method. In case of missing values, a value corresponding to the mean of the diet group was assigned (1.8% of data points were missing). AUC data was analyzed in an ANCOVA model (referred to as model B) with subject as a random factor and diet and occasion as fixed factors and baseline (time 0) as a covariate, as well as diet x time and diet x occasion interactions. Interaction terms and occasion were removed when not significant (*p* ≥ 0.05).

The energy intake during lunch and the palatability scores were analyzed using the same procedure as model B, but without the inclusion of a baseline covariate.

The factorial design, evaluating the effect of varying content of rye and varying content of sourdough, was analyzed as a secondary exploratory analysis by replacing “diet” with “sourdough (high vs. low content)” and “rye (high vs. low content)” in model A and B. The reference bread and medium sourdough/medium rye bread (MS/MR) were excluded from the analysis of the factorial design.

Tukey adjustment for multiple comparisons was applied to all results and *p* < 0.05 was considered statistically significant. No formal power calculation was conducted for this investigation, since previous studies have estimated the number of subjects needed under similar conditions [26]. Statistical analyses were carried out using SAS (version 9.4, SAS Institute Inc., Cary, NC, USA). Figures were made using GraphPad prism (version 7.02, GraphPad Software Inc., La Jolla, CA, USA).

## 3. Results

Eight men and 15 women were included in the study and all participants completed all parts of the study, with the exception of one subject who did not participate in the lunch test after consuming bread type mid/mid for reasons unrelated to the study (Figure S1). The participants had a mean age of 32 years (sd: 9.8, range 23–63) and a mean BMI of 22.5 (sd: 2.7, range: 17.3–29.3).

During the study, it was discovered that two subjects had BMI outside the inclusion criteria (17.3 and 29.3 kg/m^2^, respectively). All the statistical analyses were conducted both with and without the inclusion of these two subjects. For the majority of the outcomes this did not qualitatively affect the outcome of the analysis and the two participants were therefore included to increase power, but it is clearly specified throughout the manuscript in those cases when exclusion of the subjects significantly affected the outcome of the statistical tests.

### 3.1. Microscopy

**Figure 1:**
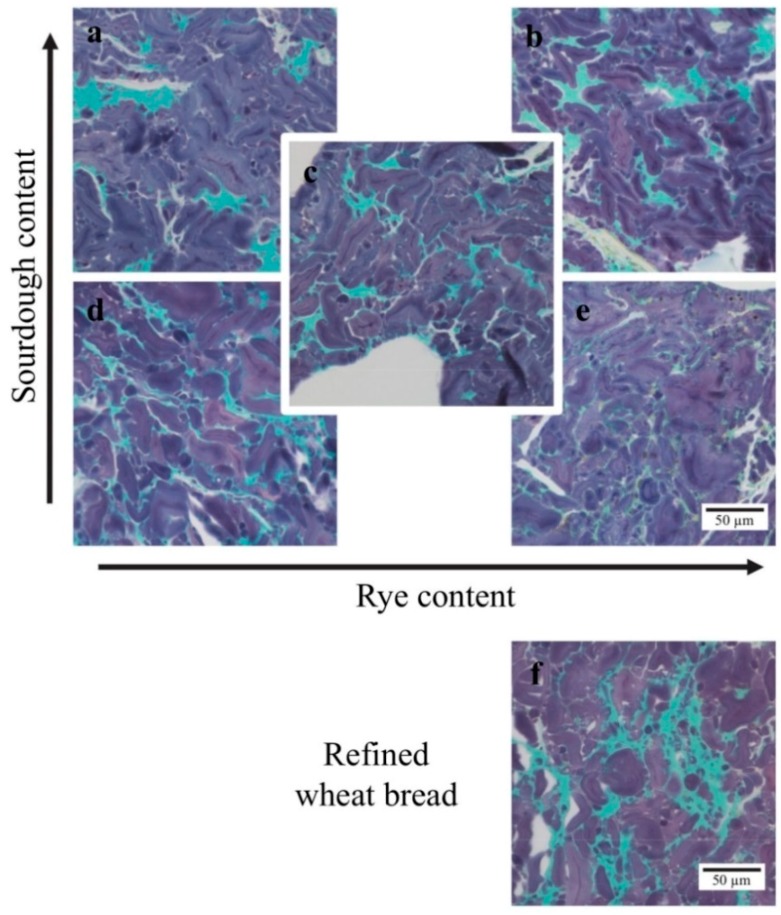
LM micrographs of samples stained with Fast Green and Lugol’s solution. Protein is colored green and starch purple/violet. Amylopectin appears beige/brown and amylose blue. The breads shown are: (**a**) high sourdough/low rye, (**b**) high sourdough/high rye, (**c**) medium sourdough/medium rye, (**d**) low sourdough/low rye, (**e**) low sourdough/high rye, (**f**) refined wheat control bread.

Protein (colored green) was observed to form continuous networks surrounding the starch (colored purple/violet) in all breads (Figure 1). In the breads with low sourdough content, the protein formed a continuous fine stranded network surrounding individual starch granules in the matrix. With increasing sourdough content, the protein networks became less extensive and protein occurred in larger aggregates in the matrix. The protein network in the refined wheat bread was more similar to the protein network in the breads with medium and high sourdough content than in the breads with low content of sourdough. The structure of the starch granules appeared similar in all five sourdough rye breads.

**Figure 2:**
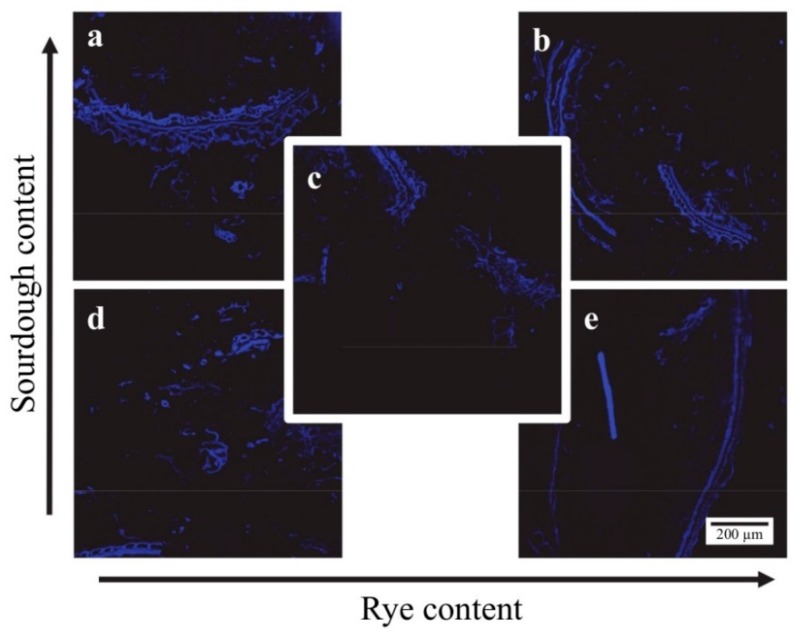
Fluorescence micrographs stained with calcoflour white. Beta-glucans are seen as blue. (**a**) high sourdough/low rye, (**b**) high sourdough/high rye, (**c**) medium sourdough/medium rye, (**d**) low sourdough/low rye, (**e**) low sourdough/high rye.

There was no apparent difference between any of the sourdough rye breads with regards to cell wall structure as visualized by staining of β-glucan with calcofluor (Figure 2).

### 3.2. Appetite

**Figure 3:**
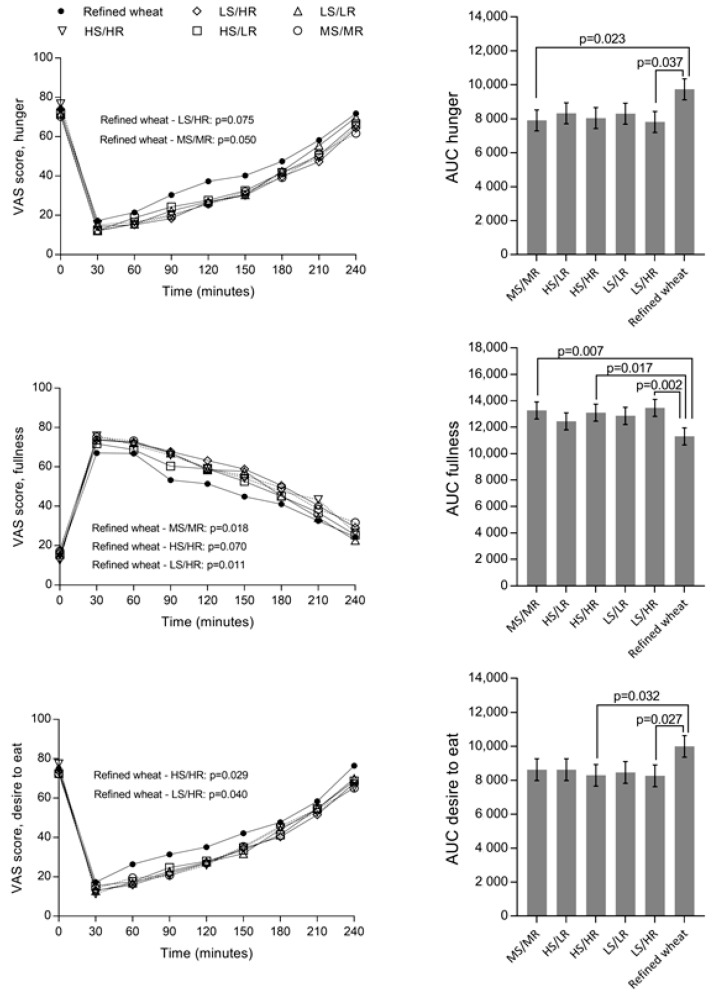
Appetite ratings following consumption of breads with varying content of rye and sourdough and a refined wheat control as part of a breakfast meal (**left**). AUC of appetite measures (**right**) are baseline adjusted least square means with standard error of mean. *p*-values are the result of pairwise comparisons between diets in an ANCOVA, after Tukey adjustment for multiple comparisons. *p*-values in the left column stems from pairwise comparisons in a repeated measures ANCOVA, after Tukey adjustment for multiple comparisons. Abbreviations: VAS, visual analogue scale; AUC, area under the curve; MS/MR, medium sourdough/medium rye; HS/LR, high sourdough/low rye; HS/HR, high sourdough/high rye; LS/LR, low sourdough/low rye; LS/HR, low sourdough/high rye.

Diet had an effect on both fullness (model A: *p* = 0.008; model B: *p* = 0.002), desire to eat (model A: *p* = 0.027; model B: *p* = 0.027) and hunger, although only borderline significant for model A (model A: *p* = 0.058; model B: *p* = 0.023) (Figure 3).

Pairwise comparisons showed that medium sourdough/medium rye (MS/MR), high sourdough/high rye (HS/HR), and low sourdough/high rye (LS/HR) resulted in higher fullness rating, compared to the reference bread. The subjects’ desire to eat were lower after consumption of HS/HR and LS/HR compared to reference bread. MS/MR and LS/HR resulted in lower hunger ratings, compared to refined bread, according to model B, while there was only a tendency according to model A (Figure 3).

Excluding the two subjects with BMI outside the desired range did not alter the conclusions with regards to hunger and fullness, but reduced the effect of diet on desire to eat to a tendency (model A: *p* = 0.051; model B: *p* = 0.057).

**Figure 4:**
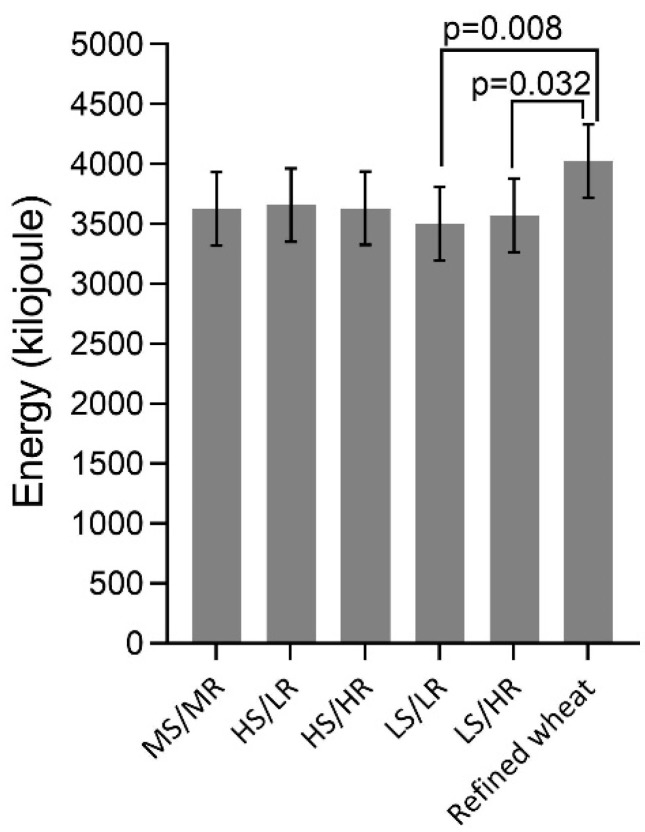
Energy intake at an ad libitum lunch meal 4 h after consumption of breads with varying content of rye and sourdough and a refined wheat control as part of a breakfast meal. *p*-values are the result of pairwise comparisons between diets in an ANCOVA model, after Tukey adjustment for multiple comparisons. Abbreviations: MS/MR, medium sourdough/medium rye; HS/LR, high sourdough/low rye; HS/HR, high sourdough/high rye; LS/LR, low sourdough/low rye; LS/HR, low sourdough/high rye.

Analysis of the amount of energy consumed in the ad libitum lunch test revealed an effect of diet (*p* = 0.032), with a 0.5 MJ reduction in energy intake following consumption of LS/LR and LS/HR at breakfast, compared to the reference bread (Figure 4). Removing the two subjects with BMI outside the desired range from the analysis, reduced the effect on energy intake following LS/HR and it was no longer different from the reference bread (mean difference 0.3 MJ, *p* = 0.319).

Analysis of the factorial design revealed no effect of rye or sourdough on appetite response.

### 3.3. Palatability

Ratings of taste, smell, appearance and overall impression did not differ between any of the test breads. Evaluation of the factorial design showed that increased content of sourdough (*p* = 0.013) and possibly also rye (*p* = 0.065) increased the off taste rating (data not shown).

## 4. Discussion

The current study showed no effect of sourdough on satiety response or overall taste rating, although the breads with highest sourdough content were perceived as having stronger off taste than the breads with lower sourdough content. The lack of differential overall taste response could be due to the study being conducted in Sweden where sourdough and rye are commonly consumed, and the distinct taste of sourdough and rye are familiar to participants. Therefore, it cannot be assumed that taste scores should be similar between test breads in a different cultural setting.

It has previously been shown that addition of acid to bread increased the satiety response and hypothesized that the effect could be mediated by lowering the gastric emptying rate, caused by the addition of acid to bread dough or soaking of bread in vinegar before consumption [13,15,27]. In the present study, the amount of sourdough in the test breads was high (up to 51% of dough weight), which is substantially higher than what is found in many commercial sourdough breads, which resulted in a pH which is in the lower range compared with commercially available breads in Sweden and to what is generally accepted by consumers [28]. But despite the high amount of sourdough, acetic acid was present in much lower concentration in the test breads (0.04–0.16 g/serving) in comparison with e.g., the model bread used by Östman et al. [15] (1.1–1.7 g/serving) and might, in fact, be too low to have a significant effect on gastric emptying. It seems highly unlikely that an acid content similar to the breads with added vinegar could be achieved without compromising the sensory qualities of the bread.

The lack of effect of sourdough on satiety and hunger in the present study could be due to the comparatively low acid content. However, we argue that it could also be due to the effects of sourdough on the structure of the final product, counteracting a potential effect of decreased pH on the gastric emptying rate. The observed structural differences with more aggregated proteins with increased sourdough content, may be related to the increased acidity of the dough. A more extensive protein network has been suggested to lead to smaller particles after gastric digestion [11], likely to be emptied faster and subsequently more rapidly digested in the small intestine, leading to higher postprandial glucose and insulin responses [11,29]. It is, however, also possible that the protein could act as a cohesive network in the masticated bolus which would need to be digested prior to emptying leading to slower disintegration during the gastric phase, but it is unknown if this would be sufficient to affect satiety. Furthermore, the increased portion of rye supplied by the sourdough could result in overall more degraded arabinoxylan, since the activity of arabinoxylan degrading enzymes increases at lower pH, with an optimum around 4.5 [30]. The ability of polymers, including arabinoxylan, to induce viscosity is highly related to concentration and molecular weight. Degradation could therefore lead to reduced viscosity and increased diffusion and absorption rates of nutrients [31]. Consequently, sourdough fermentation may in this respect have undesirable impacts on biological processes related to satiety mechanisms with downstream effects on health, such as weight management. The fact that the degradation of fibers could not be observed in the fluorescence micrographs is likely due to the intense labeling achieved with calcofluor white and the limited spatial resolution with the method used for visualization.

Rye has previously been shown to increase fullness and reduce hunger compared to refined wheat [6,7,32,33], and therefore it was hypothesized that increasing the amount of rye in the breads would improve the satiety response. However, only the breads with the highest content of rye differed from the refined wheat bread in terms of appetite response, while the breads with low rye content did not differ from either the refined wheat bread or the breads with higher rye content. In previous studies where a positive effect of rye on appetite sensation have been shown, refined soft wheat bread, crisp bread and porridge have been used as controls [32,33,34], and it cannot be ruled out that the results are to some extent confounded by the different matrices in which rye and wheat were served, including differences in portions size. It has previously been shown that ingested volume affects subjective appetite ratings [35], and variations in portions size can be a challenge when attempting to provide meals standardized for energy, which is a common practice in studies of postprandial appetite sensation. A strength of the current study is the uniformity of the portion sizes, which minimized the potential effect of variations in volume and portions size. Especially the uniformity of the rye breads, which allowed for blinding of the participants, is considered a great strength for achieving reliable subjective responses such as appetite scores. It should be noted that the design of the study did control the potential influence of menstrual cycle on appetite in female participants, which may have influenced the results [36]. Furthermore, the sample size did not allow for well powered stratified analyses that could have revealed potential sex differences in appetite response and food intake. In future studies it could be relevant to include measurements of appetite hormones and glucose metabolism, in order to support the subjective appetite ratings and to allow for deeper exploration of the potential mechanisms behind differences in appetite response.

The lack of a dose-response effect of rye content on appetite response might be at least partially explained by the relatively small difference in rye content, with the highest being 48% (30.7 g/serving) of the dough weight to the lowest being 35% (22.5 g/serving) of the flour weight. In retrospect, the range between high and low rye content could have been larger to better investigate potential dose/response effects of rye on appetite responses. Though the amount of sourdough in the refined wheat bread was small, it cannot be ruled out that it may have affected the results, and in future studies it might be favorable to produce a wheat bread without addition of sourdough, to allow for more controlled conditions.

## 5. Conclusions

In conclusion, this study does not support an effect of sourdough on appetite response, despite a generally high and variable sourdough content among the rye breads. We suggest that the observed lack of effect may be due to comparably low content of organic acids, insufficient to affect gastric emptying and subsequent glycaemia and appetite. It could also be attributed to structural changes in the bread when increasing the amount of sourdough, counteracting potential direct pH-effects on gastric emptying. Moreover, the different amounts of rye did not affect appetite responses, possibly as a consequence of a relatively small range in amounts of rye in the tested breads.

## Figures and Tables

**Table 1:** Rye bread recipes and design.

Rye Bread Sourdough/Rye	Rye Sourdough ^a^	Rye Flour	Rye Total ^b^	Wheat Flour	Total Water ^c^	% Sourdough of Total Dough	% Rye Flour of Total Flour
1. MS/MR	1250	500	1000	900	1570	30	42
2. HS/LR	2125	0	850	1050	1540	51	35
3. HS/HR	2125	300	1150	750	1540	51	48
4. LS/LR	375	700	850	1050	1600	9	35
5. LS/HR	375	1000	1150	750	1750	9	48

^a^ Sourdough consisting of 40:60 whole grain rye flour:water. ^b^ Sum of whole grain rye flour added and included in sourdough. ^c^ Sum of water added and included in sourdough. Abbreviations: MS/MR, medium sourdough/medium rye; HS/LR, high sourdough/low rye; HS/HR, high sourdough/high rye; LS/LR, low sourdough/low rye; LS/HR, low sourdough/high rye.

**Table 2:** Composition, energy content, pH and acids of breads per serving (100 g).

	MS/MR	HS/LR	HS/HR	LS/LR	LS/HR	Refined Wheat Bread
Protein (g)	7.1	7.2	6.9	7.3	6.3	7.5
Fat (g)	2.2	2.2	2.2	2.2	1.9	3.9
Starch (g)	41.2	41.9	39.9	40.4	39.8	41.9
Total fiber ^a^ (g)	7.0	6.0	7.2	6.8	8.3	3.6
Soluble fiber (g)	2.3	2.0	2.5	2.1	2.7	1.6
Insoluble fiber (g)	4.7	4.0	4.7	4.7	5.5	3.0
Ash (g)	1.7	1.6	1.7	1.7	1.5	1.4
Water (g)	35.0	35.7	36.1	35.8	35.7	38.0
Energy (kJ) ^b^	959	963	934	946	920	992
pH	4.4	4.2	4.2	5.2	5.3	5.0
Acid equivalents ^c^	10.3	11.5	12.5	6.3	6.6	4.3
Lactic acid (g)	0.81	0.81	0.89	0.4	0.36	0.27
Acetic acid (g)	0.13	0.14	0.16	0.07	0.04	0.04
Rye per serving (g)	27.3	22.5	30.7	22.5	30.1	0

^a^ Fiber content as analyzed by the Uppsala method with inclusion of fructans. ^b^ Energy content was calculated using a conversion factor of 37 kJ/g for fat, 17 kJ/g for proteins and starch and 8 kJ/g for fiber. ^c^ Acid equivalents, expressed as the amount of 0.1 mol/L sodium hydroxide (NaOH) consumed in mL/10 g bread dry wt. Abbreviations: MS/MR, medium sourdough/medium rye; HS/LR, high sourdough/low rye; HS/HR, high sourdough/high rye; LS/LR, low sourdough/low rye; LS/HR, low sourdough/high rye.

## Supplementary Materials

The following are available online at http://www.mdpi.com/2072-6643/10/11/1594/s1, Figure S1: Consort flow diagram.

## References

[1] Caballero B. (2007). The Global Epidemic of Obesity: An Overview. Epidemiol. Rev..

[2] James P.T., Rigby N., Leach R. (2004). The obesity epidemic, metabolic syndrome and future prevention strategies. Eur. J. Cardiovasc. Prev. Rehabil..

[3] Van Kleef E., Van Trijp J.C.M., Van Den Borne J.J.G.C., Zondervan C. (2012). Successful development of satiety enhancing food products: Towards a multidisciplinary agenda of research challenges. Crit. Rev. Food Sci. Nutr..

[4] Mela D.J. (2006). Eating for pleasure or just wanting to eat? Reconsidering sensory hedonic responses as a driver of obesity. Appetite.

[5] Blundell J. (2010). Making claims: Functional foods for managing appetite and weight. Nat. Rev. Endocrinol..

[6] Isaksson H., Fredriksson H., Andersson R., Olsson J., Aman P. (2009). Effect of rye bread breakfasts on subjective hunger and satiety: A randomized controlled trial. Nutr. J..

[7] Rosén L.A.H., Östman E.M., Shewry P.R., Ward J.L., Andersson A.A.M., Piironen V., Lampi A.-M., Rakszegi M., Bedö Z., Björck I.M.E. (2011). Postprandial Glycemia, Insulinemia, and Satiety Responses in Healthy Subjects after Whole Grain Rye Bread Made from Different Rye Varieties. 1. J. Agric. Food Chem..

[8] Clark M.J., Slavin J.L. (2013). The effect of fiber on satiety and food intake: A systematic review. J. Am. Coll. Nutr..

[9] Parada J., Aguilera J.M. (2011). Review: Starch matrices and the glycemic response. Food Sci. Technol. Int..

[10] Juntunen K.S., Laaksonen D.E., Poutanen K.S., Niskanen L.K., Mykkanen H.M. (2003). High-fiber rye bread and insulin secretion and sensitivity in healthy postmenopausal women. Am. J. Clin. Nutr..

[11] Nordlund E., Katina K., Mykkänen H., Poutanen K. (2016). Distinct Characteristics of Rye and Wheat Breads Impact on their in Vitro Gastric Disintegration and in Vivo Glucose and Insulin Responses. Foods.

[12] Hugenholtz J. (2013). Traditional biotechnology for new foods and beverages. Curr. Opin. Biotechnol..

[13] Liljeberg H., Björck I. (1998). Delayed gastric emptying rate may explain improved glycaemia in healthy subjects to a starchy meal with added vinegar. Eur. J. Clin. Nutr..

[14] Delzenne N., Blundell J., Brouns F., Cunningham K., De Graaf K., Erkner A., Lluch A., Mars M., Peters H.P.F., Westerterp-Plantenga M. (2010). Gastrointestinal targets of appetite regulation in humans. Obes. Rev..

[15] Östman E., Granfeldt Y., Persson L., Björck I. (2005). Vinegar supplementation lowers glucose and insulin responses and increases satiety after a bread meal in healthy subjects. Eur. J. Clin. Nutr..

[16] Liljeberg H.G., Lönner C.H., Björck I.M. (1995). Sourdough fermentation or addition of organic acids or corresponding salts to bread improves nutritional properties of starch in healthy humans. J. Nutr..

[17] Darzi J., Frost G.S., Robertson M.D. (2012). Effects of a novel propionate-rich sourdough bread on appetite and food intake. Eur. J. Clin. Nutr..

[18] Bondia-Pons I., Nordlund E., Mattila I., Katina K., Aura A.-M., Kolehmainen M., Orešič M., Mykkänen H., Poutanen K. (2011). Postprandial differences in the plasma metabolome of healthy Finnish subjects after intake of a sourdough fermented endosperm rye bread versus white wheat bread. Nutr. J..

[19] Tholin S., Rasmussen F., Tynelius P., Karlsson J. (2005). Genetic and environmental influences on eating behavior: The Swedish Young Male Twins Study. Am. J. Clin. Nutr..

[20] Hellström A.M., Vázques-Juárez R., Svanberg U., Andlid T.A. (2010). Biodiversity and phytase capacity of yeasts isolated from Tanzanian togwa. Int. J. Food Microbiol..

[21] Theander O., Aman P., Westerlund E., Andersson R., Pettersson D. (1995). Total dietary fiber determined as neutral sugar residues, uronic acid residues, and Klason lignin (the Uppsala method): Collaborative study. J. AOAC Int..

[22] McCleary B.V., Murphy A., Mugford D.C. (2000). Measurement of total fructan in foods by enzymatic/spectrophotometric method: Collaborative study. J. AOAC Int..

[23] McCleary B.V., Monaghan D.A. (1997). Measurement of total starch in cereal products by amyloglucosidase-α-amylase method: Collaborative study. J. AOAC Int..

[24] Johansson D.P., Lee I., Risérus U., Langton M., Landberg R. (2015). Effects of unfermented and fermented whole grain rye crisp breads served as part of a standardized breakfast, on appetite and postprandial glucose and insulin responses: A randomized cross-over trial. PLoS ONE.

[25] Whybrow S., Stephen J.R., Stubbs R.J. (2006). The evaluation of an electronic visual analogue scale system for appetite and mood. Eur. J. Clin. Nutr..

[26] Flint A., Raben A., Blundell J.E., Astrup A. (2000). Reproducibility, power and validity of visual analogue scales in assessment of appetite sensations in single test meal studies. Int. J. Obes. Relat. Metab. Disord..

[27] Darwiche G., Ostman E.M., Liljeberg H.G., Kallinen N., Björgell O., Björck I.M., Almér L.O. (2001). Measurements of the gastric emptying rate by use of ultrasonography: Studies in humans using bread with added sodium propionate. Am. J. Clin. Nutr..

[28] Sandvik P., Marklinder I., Nydahl M., NAEs T., Kihlberg I. (2016). Characterization of Commercial Rye Bread Based on Sensory Properties, Fluidity Index and Chemical Acidity. J. Sens. Stud..

[29] Hellström P.M., Grybäck P., Jacobsson H. (2006). The physiology of gastric emptying. Best Pract. Res. Clin. Anaesthesiol..

[30] Rasmussen C.V., Boskov Hansen H., Hansen Å., Melchior Larsen L. (2001). pH-, Temperature- and Time-dependent Activities of Endogenous Endo-β-d-Xylanase, β-d-Xylosidase and α-l-Arabinofuranosidase in Extracts from Ungerminated Rye (*Secale cereale* L.) Grain. J. Cereal Sci..

[31] Shelat K.J., Vilaplana F., Nicholson T.M., Wong K.H., Gidley M.J., Gilbert R.G. (2010). Diffusion and viscosity in arabinoxylan solutions: Implications for nutrition. Carbohydr. Polym..

[32] Rosén L.A., Ostman E.M., Björck I.M. (2011). Effects of cereal breakfasts on postprandial glucose, appetite regulation and voluntary energy intake at a subsequent standardized lunch; focusing on rye products. Nutr. J..

[33] Isaksson H., Tillander I., Andersson R., Olsson J., Fredriksson H., Webb D.-L., Åman P. (2012). Whole grain rye breakfast—Sustained satiety during three weeks of regular consumption. Physiol. Behav..

[34] Forsberg T., Åman P., Landberg R. (2014). Effects of whole grain rye crisp bread for breakfast on appetite and energy intake in a subsequent meal: Two randomised controlled trails with different amounts of test foods and breakfast energy content. Nutr. J..

[35] Burton P., Lightowler H.J. (2006). Influence of bread volume on glycaemic response and satiety. Br. J. Nutr..

[36] Hirschberg A.L. (2012). Sex hormones, appetite and eating behaviour in women. Maturitas.

