# Supplementary figures and images for: Appetite and Subsequent Food Intake Were Unaffected by the Amount of Sourdough and Rye in Soft Bread—A Randomized Cross-Over Breakfast Study
Source: Nutrients. 2018 Oct 30;10(11):1594. doi: 10.3390/nu10111594 (PMC6266039; doi:10.3390/nu10111594)

**Supplemental figure S1: CONSORT flow diagram**

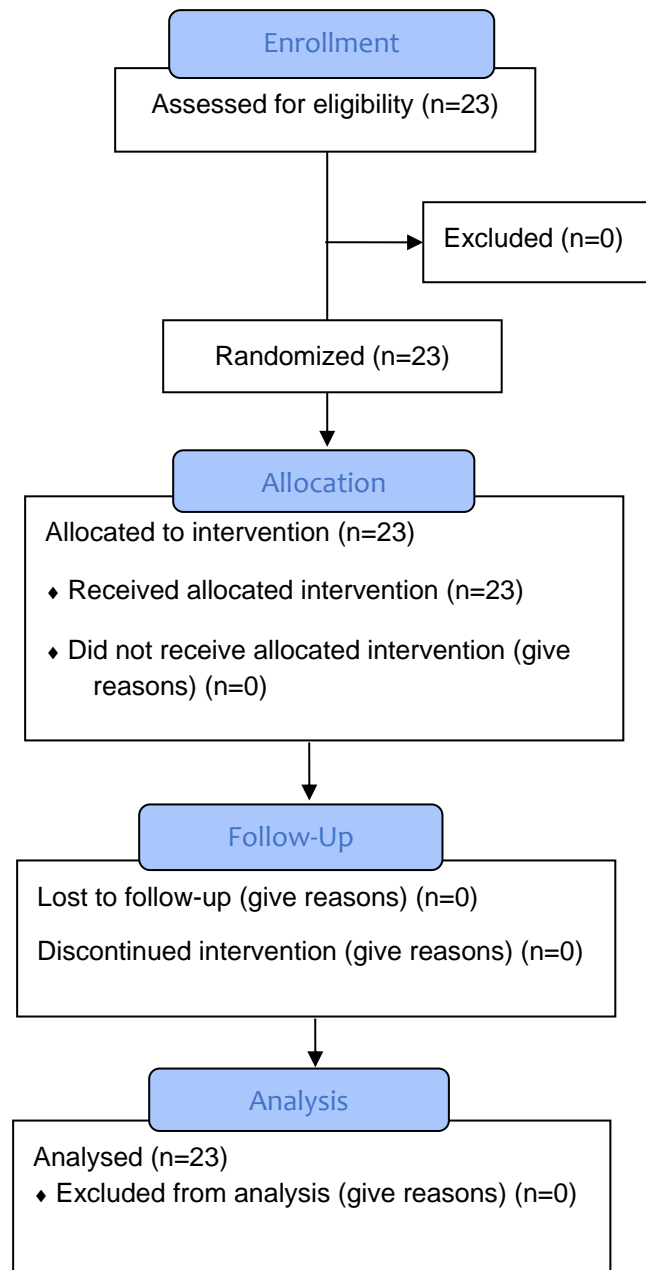

Supplement: Supplementary file 1 [file nutrients-10-01594-s001.pdf]
